# A Novel Hierarchical Porous 3D Structured Vanadium Nitride/Carbon Membranes for High-performance Supercapacitor Negative Electrodes

**DOI:** 10.1007/s40820-018-0217-1

**Published:** 2018-07-13

**Authors:** Yage Wu, Yunlong Yang, Xiaoning Zhao, Yongtao Tan, Ying Liu, Zhen Wang, Fen Ran

**Affiliations:** 1 State Key Laboratory of Advanced Processing and Recycling of Non-ferrous Metals, Lanzhou University of Technology, Lanzhou, 730050 People’s Republic of China; 2 School of Material Science and Engineering, Lanzhou University of Technology, Lanzhou, 730050 People’s Republic of China

**Keywords:** Supercapacitors, Vanadium nitride/carbon, 3D network, Hierarchical porous structure

## Abstract

Transition-metal nitrides exhibit wide potential windows and good electrochemical performance, but usually experience imbalanced practical applications in the energy storage field due to aggregation, poor circulation stability, and complicated syntheses. In this study, a novel and simple multi-phase polymeric strategy was developed to fabricate hybrid vanadium nitride/carbon (VN/C) membranes for supercapacitor negative electrodes, in which VN nanoparticles were uniformly distributed in the hierarchical porous carbon 3D networks. The supercapacitor negative electrode based on VN/C membranes exhibited a high specific capacitance of 392.0 F g^−1^ at 0.5 A g^−1^ and an excellent rate capability with capacitance retention of 50.5% at 30 A g^−1^. For the asymmetric device fabricated using Ni(OH)_2_//VN/C membranes, a high energy density of 43.0 Wh kg^−1^ at a power density of 800 W kg^−1^ was observed. Moreover, the device also showed good cycling stability of 82.9% at a current density of 1.0 A g^−1^ after 8000 cycles. This work may throw a light on simply the fabrication of other high-performance transition-metal nitride-based supercapacitor or other energy storage devices.

**Electronic supplementary material:**

The online version of this article (10.1007/s40820-018-0217-1) contains supplementary material, which is available to authorized users.

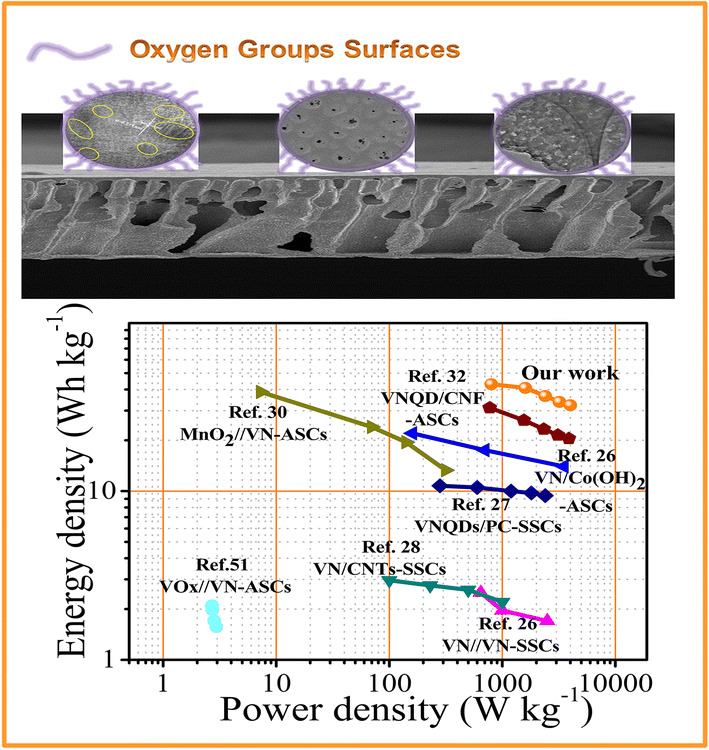

## Highlights

- A novel and simple multi-phase polymeric strategy was used to fabricate hierarchical porous 3D structured vanadium nitride/carbon (VN/C) membranes.
- The supercapacitor negative electrodes based on VN/C membranes exhibited a high specific capacitance of 392.0 F g^−1^ at 0.5 A g^−1^ and an excellent rate capability with capacitance retention of 50.5% at 30 A g^−1^.
- The asymmetric device fabricated using Ni(OH)_2_/VN/C membranes has a high energy density of 43.0 Wh kg^−1^ at a power density of 800 W kg^−1^ and good cycling stability of 82.9% at 1.0 A g^−1^ after 8000 cycles.

## Introduction

With the rapid development of the global economy and growing population, energy, as a pillar of modern civilization, has received increasing attention. From the development of clean fuel such as wind power, solar energy, water energy, and tidal power, the tension between rising energy demand and environmental protection is easing [1–4]. However, the existing energy output of clean-fuel technology is subject to discontinuity and variable environmental factors. For efficient use of renewable energy, it is important to develop high-efficient and stable energy storage devices. Supercapacitors, also called electrochemical capacitors, represent environment-friendly and irreplaceable energy storage devices compared to traditional capacitors and rechargeable batteries. Supercapacitors can achieve greater energy and power densities than conventional energy storage devices [5–7].

Supercapacitors can be classified into electrical double-layer capacitors (EDLCs) and pseudocapacitors [8–12]. Considering their high-power densities, long cycle life, and fast charging/discharging rate, EDLCs have been widely used in commercial supercapacitor applications [13]. Typical electrode materials for EDLCs are carbon-based and include activated carbon, carbon black, carbon onions, carbon nanotubes, and graphene [14–18]. However, a crucial limitation of EDLCs is their low energy densities of approximately 5–15 Wh kg^−1^, primarily due to the fast sorption and desorption of ions on the carbon-based electrode [19]. Pseudocapacitors chemically store charge through fast and reversible redox reactions at the electrode interface. Electrode materials for pseudocapacitors should exhibit considerable capacity but are typically constrained by poor conductivity and stability. Most electrode materials consist of metal oxides and conducting polymers such as iron oxide [13], manganese oxide [20, 21], vanadium nitride [22, 23], tungsten nitride [24], and polyaniline [25].

To increase the energy storage and stability of the two-electrode materials, it is necessary to combine carbon-based materials with high-capacitance pseudocapacitive materials [6, 16, 20–23]. Vanadium nitride (VN) has been shown to be a suitable candidate to improve the specific capacitance and energy density of negative electrode materials because of its excellent electrical conductivity as well as its wide and electrochemically stable potential window [26–36]. It has been reported that coating carbon on the VN surface largely improved its stability during electrochemical reaction [37]. However, two major obstacles hinder the efficient fabrication of VN and carbon electrodes. The first is controlling the uniform distribution of VN nanoparticles in carbon matrix to prevent VN aggregation. The other problem is improving the infiltration of the carbon surface without affecting the VN state and its distribution within the internal holes of the material [38]. In addition, the synthetic routes for nanocomposites of VN and carbon remain limited due to rather time-consuming, costly, and complex fabrication methods which include solution adsorption, chemical vapor deposition, laser atomic layer deposition, and electrospinning. Therefore, developing effective synthetic methods is particularly important for achieving novel supercapacitors with improved performance.

In this study, a novel and simple synthetic method was used to fabricate electrode membrane materials in which VN nanoparticles are uniformly incorporated into a 3D carbon matrix. Solvent exchange, PEG immigration, and self-assembly of the tri-block copolymer PAN-b-PMMA-b-PAN were applied to form an asymmetric 3D polymer membrane with hierarchical porous nanostructure. The electrochemical performances including specific capacitance, rate ability, and energy density based on the 3D VN/C membranes electrode and the supercapacitor device were investigated.

## Experimental

### Chemicals

Vanadyl acetylacetonate was purchased from Sinopharm Chemical Reagent Co. Ltd. and used as received without further treatment. Polyethylene glycol (PEG, M_*w*_= 400) was obtained from Aladdin (Shanghai, China). PAN was prepared by solution polymerization as previously described [39, 40]. The tri-block copolymer (BCP) PAN-*b*-PMMA-*b*-PAN was synthesized by reversible addition fragmentation chain (RAFT) polymerization as previously described [40]. All other chemicals (analytical grade) were obtained from Sinopharm Chemical Reagents Co. Ltd, China, and used without further purification.

### Preparation of the V Source and Uniformly Distributed Polymer Membrane

Vanadyl acetylacetonate (0.5 g), PAN (1.5 g), PEG (0.5 g), and BCP (0.4 g) were dissolved in *N,N*-dimethylformamide (DMF, 11.2 g) with continuous stirring at 60 °C until a dark green homogeneous solution was obtained, which was subsequently used as casting solution. The material of vanadyl acetylacetonate/multi-phase polymeric membranes (I) was prepared by spin coating the casting solution on glass at 20 °C. Immediately, the membrane was submerged in deionized water as coagulation bath and subsequently the cured membrane was transferred to an air atmosphere at 40 °C to remove the residual water and solvent in the membrane. A casting solution without PEG and BCP was prepared to fabricate vanadyl acetylacetonate/polymeric membranes (II) using the same process as for vanadyl acetylacetonate/multi-phase polymeric membranes (I).

### Synthesis of the Hierarchical Porous Vanadium Nitride/Carbon (VN/C) Membranes

The V/P-M membrane was first heated at 250 °C for 2 h under air flow and then sintered at 800 °C for 1.5 h under a mixed gas of NH_3_:N_2_ = 3:2. After cooling to room temperature, the hierarchical porous VN/C (I) and VN/C (II) were obtained from vanadyl acetylacetonate/multi-phase polymeric membranes (I) and vanadyl acetylacetonate/polymeric membranes (II), respectively.

### Materials Characterization

The microscopic morphologies of the samples were characterized by field emission scanning electron microscopy (FE-SEM, JSM-6701F, JEOL, Japan) and transmission electron microscopy (TEM, JEOL, JEM-2010, Japan). The crystal structure was identified by X-ray diffraction (XRD, D/MAX 2400, Japan) with Cu Kα radiation (*k* = 1.5418 Å) operating at 40 kV and 60 mA. The N_2_ adsorption–desorption isotherms of samples were measured at 77 K using an ASAP 2460 (Micromeritics, USA) instrument to measure the specific surface area. The specific surface area was calculated using the Brunauer–Emmett–Teller plot of the nitrogen adsorption isotherm. Non-local density functional theory (NLDFT) model was adopted to analyze the pore size distribution of samples (calculation model: slit/cylindrical pore, NLDFT equilibrium model). X-ray photoelectron spectroscopy (XPS) analysis was performed using a PerkinElmer PHI ESCA system with Al K*α* (1486.6 eV) as the X-ray source. The electrical conductivity of the samples was determined using a four-point probe (RTS-9).

### Electrochemical Measurements

All electrochemical measurements including cyclic voltammetry (CV), galvanostatic charging/discharging (GCD), and electrochemical impedance spectroscopy (EIS) were taken using an electrochemical working station (CHI660E, Shanghai, China) in 6 M KOH aqueous electrolyte. A three-electrode system including the as-prepared active material as the working electrode, platinum foil electrode as the counter electrode, and saturated calomel electrode (SCE) as the reference electrode was used to evaluate electrochemical performance. The working electrode was prepared using mixed active material, conducting graphite, acetylene black, and a poly(tetrafluoroethylene) emulsion at a weight ratio of 80:7.5:7.5:5, then pressed onto a 1-cm^2^ nickel foam current collector at 10 MPa, and dried at 60 °C for 8 h. The mass loading of the active material was 4 mg cm^−2^. The specific mass capacitance of the electrode based on the galvanostatic cycle test was calculated by Eq. 1,

1 $$ C = I \times \Delta t/(\Delta V \times m) $$

where *I* (A), Δ*t* (s), Δ*V* (V), and *m* (mg) are the discharging current, discharging time, voltage range during discharging, and mass of the electrode, respectively.

For the assembled supercapacitor, charge storage on the positive and negative electrodes was accurately described by *q*_+_ = *q*_−_. To balance the charge storage, the mass matching of the positive and negative electrodes was optimized by Eq. 2:

2 $$ m_{ + } /m_{ - } = \, (C_{ - } \times \Delta V_{ - } )/ \, (C_{ + } \times \Delta V_{ + } ) $$

The energy density and average power energy were calculated by Eqs. 3 and 4:

3 $$ E = {\text{CV}}^{2} /7.2 $$

4 $$ P = \, 3600E/(\Delta t) $$

where *E* (Wh kg^−1^), *C* (F g^−1^), *V* (V), *P* (W kg^−1^), and ∆t (s) are the energy density of the device, specific capacitance, potential drop during discharging, power density of the device, and discharging time, respectively.

## Results and Discussion

**Scheme 1:**
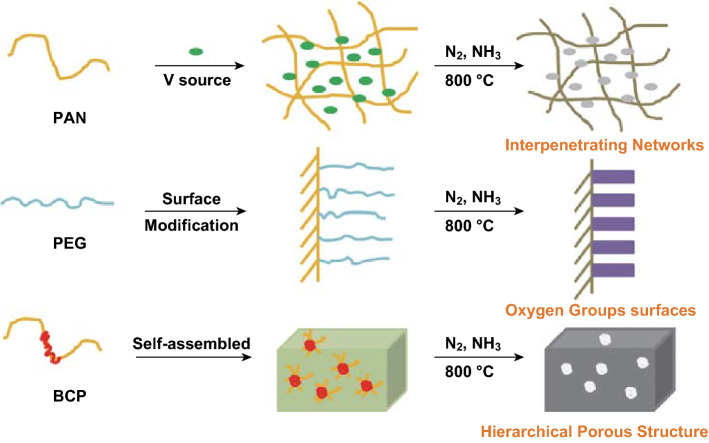
Schematic representation of the fabrication strategy for the VN/C (I)

The structure architecture of the interpenetrating carbon/vanadium nitride networks, oxygen functional group-containing surfaces, and hierarchical porous structure is schematically shown in Scheme 1. The polymer casting solution including PAN, PEG, and BCP (PAN-*b*-PMMA-*b*-PAN) was spin coated and immersed into a non-solvent water bath to induce phase separation for membrane formation. In the process, vanadyl acetylacetonate was added to the casting solution, as vanadyl acetylacetonate and PAN show good blending compatibility in DMF at a suitable temperature. We sought to determine the optimal conditions for uniform dispersion of V into the polymer system in the polymer precursor/V source hybrid system. Through thermotreatment under a NH_3_/N_2_ atmosphere, interpenetrating carbon/VN networks were obtained. In the networks, VN was dispersed uniformly and was wrapped by the carbon scaffold. During the phase process, PEG moved to the surface of the membrane, which generated oxygen-containing functional groups on the surface. The functional groups through sintering improved the infiltration of the electrode in electrolyte solution. Solvent exchange, PEG immigration, and self-assembly of BCP created an asymmetric 3D polymer membrane with hierarchical porous structure. This unique structure was preserved in the final carbon/VN material after heat treatment. Solvent exchange and PEG migration leave micropores with a variety of pore sizes. On the other hand, self-assembled PMMA blocks from BCP were used as a “sacrificed chain” and generated abundant mesopores during high-temperature carbonation. In addition, under a NH_3_ and N_2_ mixed atmosphere at 800 °C, VN/C (I) was doped with nitrogen and underwent further modification.

**Fig. 1:**
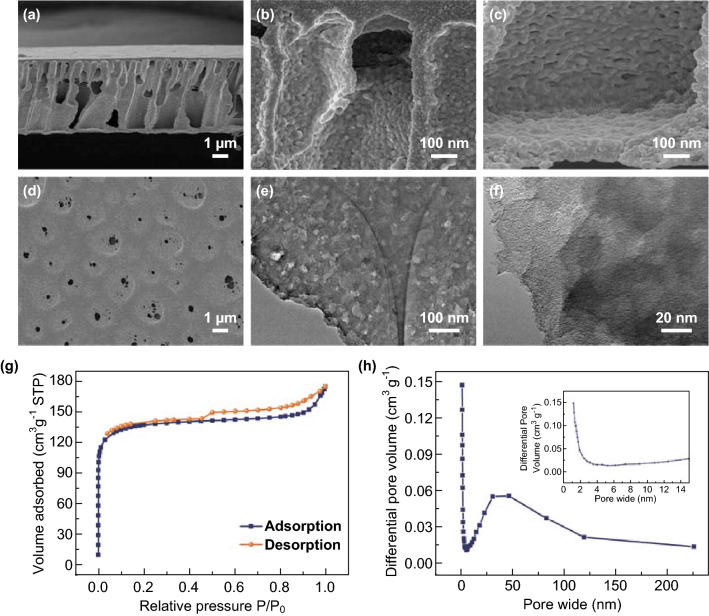
SEM images of the VN/C (I): **a**–**c** cross-sectional views and **d** surface view. **e**, **f** TEM images of the VN/C (I). **g**, **h** N_2_ adsorption–desorption isotherms and pore size distribution of the VN/C (I)

SEM, TEM, and N_2_ adsorption–desorption isotherms of VN/C (I) were used to study the morphology as well as pore structure and distribution, as shown in Fig. 1. Typical SEM cross-sectional views of the VN/C (I) sample showed a classic membrane structure formed by the liquid–liquid phase-separation method (Fig. 1a–c**)**. The representative morphology of the asymmetric membrane with a thickness of approximately 5.0 μm was observed with a regular and uniform shape and porous structure (Fig. S1). By comparing the SEM images of top and bottom sections of the VN/C (I) (Fig. 1b–c) and VN/C (II) (Fig. S1b–c), VN/C (I) exhibited more mesoporous features, as shown in the high-magnification SEM images. According to the SEM images, as shown in Fig. 1d and S2, a uniform concave size appeared in the VN/C (I) sample that was caused by the aggregation of BCP and loss of PMMA blocks during phase inversion and thermotreatment, respectively. However, the surface of the VN/C (II) sample was smooth, fine, and compact with few pores, which significantly decreased ion accumulation at its surface. In addition, from the TEM images, as shown in Fig. 1e–f and S3, abundant homogeneous mesopores approximately 40–50 nm in size were observed throughout the VN/C (I) material, but none were observed in VN/C (II). The porous structures of the membranes were also analyzed by nitrogen adsorption. As shown in Fig. 1g–h and S4, and Table S1, the VN/C (I) sample exhibited a typical type-IV curve with an average pore diameter of 1.9 nm and total pore volume of 0.24 cm^3^ g^−1^. A wide pore size distribution range from 1.7 to 250 nm was observed, which manifested as a loose network with micro-, meso-, and macropores. In comparison, the VN/C (I) sample exhibited a larger specific surface area (SSA, 523.5 m^2^ g^−1^) than that of VN/C (II) (504.2 m^2^ g^−1^), which was also much higher than previously reported VN and carbon composite materials [38, 41–44]. Notably, the pore distribution range with larger pores, formed by adding PEG and BCP to the casting solution, can act as ion buffer pools by storing electrolytes and facilitating ion transmission to improve the efficiency of the material ratio surface area.

**Fig. 2:**
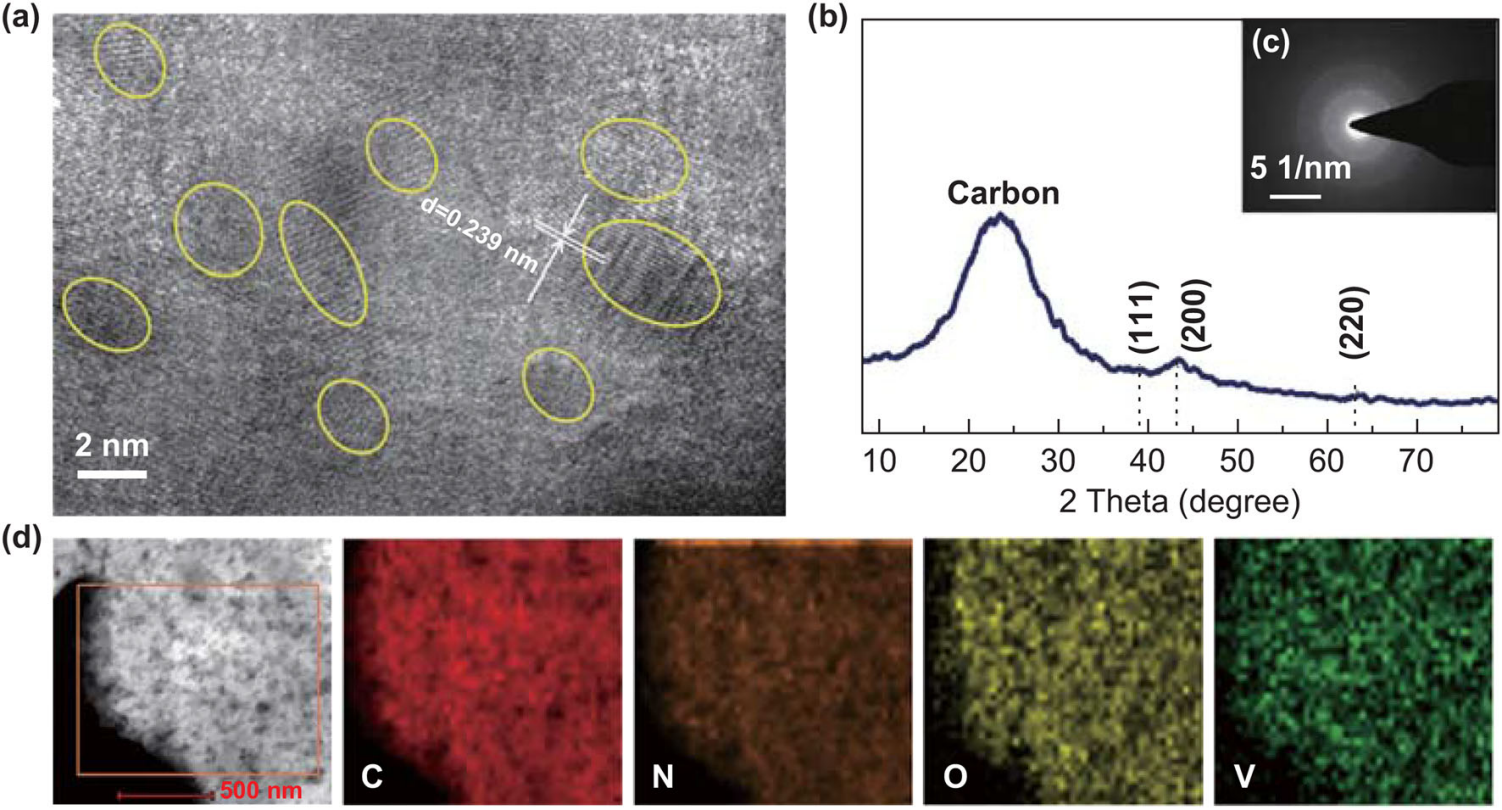
**a** HRTEM image, **b** XRD pattern, **c** selected-area electron diffraction, and **d** TEM elemental mapping images of the VN/C (I)

Through the phase-separation and thermotreatment process, an interpenetrating polymer/V-sources network was formed followed by fabrication of interpenetrating carbon/VN networks. Figure 2 shows the correlation representational data that highlight the homogeneity of the VN/C (I) sample. The high-resolution TEM image (Fig. 2a) exhibited a large number of small VN quantum dots with 5–8 nm in size, which was evenly and densely embedded in the carbon substrates. The low-crystalline nature of VN/C (I) was indicated by surface area electron diffraction (SAED) measurements (Fig. 2c). The XRD pattern of the VN/C (I) sample (Fig. 2b) exhibits a broad peak at approximately 22°, indicating the presence of amorphous carbon derived from the PAN precursor in the homopolymer or BCP. This carbon scaffold improves the electrical conductivity of the material and increases the utilization and stability of VN quantum dots. In addition, a slightly stronger peak at 43.6° and two weak peaks at approximately 37.4° and 63.4° were, respectively, ascribed to the (2 0 0), (1 1 1), and (2 2 0) diffractions of the VN (ICDD PDF 35-768) [27, 31]. The XRD results and SAED pattern indicate that the prepared VN/C (I) contained amorphous carbon and VN with low crystallinity. Figure 2d shows the TEM elemental mapping images of VN/C (I), which revealed uniform distributions of C, N, O, and V throughout the material. In conclusion, these results indicated that the VN nanoparticles were evenly distributed in the substrate material. The carbon provided active sites for electrolyte ions by preventing VN grain growth and aggregation [22, 23, 27, 32–36].

**Fig. 3:**
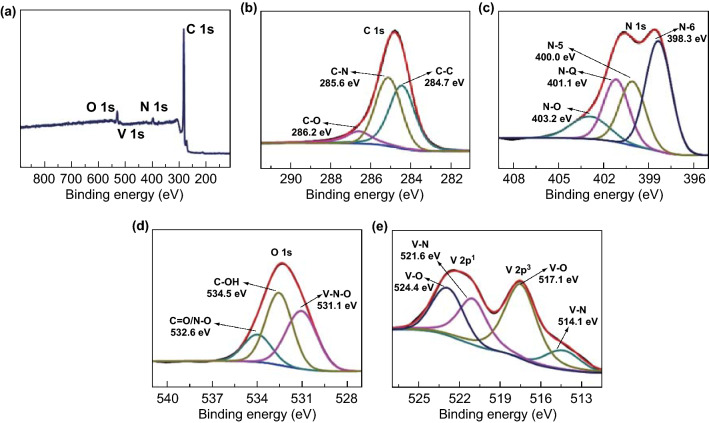
X-ray photoelectron spectra of the VN/C (I)

To further explore the surface modification, XPS of the VN/C (I) (Fig. 3) and VN/C (II) (Fig. S5) samples was performed. The overall XPS spectra show that the surface of samples consisted of C, N, V, and O, and the corresponding analytical results are summarized in Table S2. The respective proportions of C, N, V, and O in VN/C (I) and VN/C (II) were 88.0, 3.6, 2.3, 6.1 at%, and 87.8, 4.2, 2.7, 5.3 at%, respectively. Three main peaks at 284.7, 285.6, and 286.2 eV were observed in the C 1 s spectrum in Fig. 3b and were attributed to C–C, C–N, and C–O bonds, respectively [9, 13, 45, 46]. As illustrated in Fig. 3c, the N 1 s signal could be partitioned into four characteristic peaks at 398.3, 400.0, 401.1, and 403.2 eV corresponding to pyridinic N (N-6), pyrrolic N (N-5), graphitic N (N-Q), and oxygenated N (N–O), respectively [19, 46–48]. As shown in Fig. 3d, the peaks centered at 531.1, 532.6, and 534.5 eV were assigned to V–N–O, C = O/N–O, and C–OH, respectively [19, 31, 39, 40]. In addition, the peaks at approximately 514.1 and 521.6 eV typical of vanadium in the VN structure, and the peaks at 517.1 and 524.4 eV arose from the V–O bonds on the surface of VN/C (I) (Fig. 3e) [22–24, 30, 31, 49]. Comparing the C 1 s, N 1 s, and O 1 s spectra of the two samples, it is clear that more oxygen-containing groups such as C–O (20.0 at%), N–O (20.9 at%), and C–OH (29.7 at%) were present in VN/C (I) compared to the VN/C (II) sample (C–O 17.1 at%, N–O 12.3 at%, and C–OH 16.9 at%). These results indicated that PEG formed oxygen functional groups through sintering at the surface, which improved the infiltration of the VN/C (I) electrode.

**Fig. 4:**
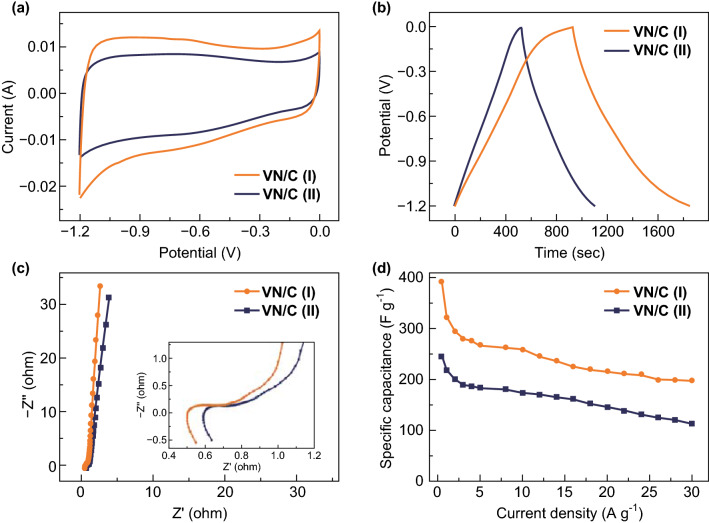
**a** CV, **b** GCD, **c** EIS curves, and **d** the specific capacitances of the VN/C (I) and VN/C (II) at different current densities (scan rate = 10 mV s^−1^ in 6.0 M KOH aqueous solution)

To investigate the electrochemical capacitive performance of the prepared samples, CV, GCD, and EIS were measured using a three-electrode system in 6 M KOH aqueous electrolyte with a SCE and Pt as the reference and counter electrodes, respectively. The CV curves of the VN/C (I) and VN/C (II) samples were obtained at a scan rate of 10 mV s^−1^ in the potential range of − 1.2–0 V, as shown in Fig. 4a. Both curves were quasi-rectangular in shape and featured broad redox humps, indicating typical double-layer capacitive behavior with Faradaic reactions [48, 50, 51]. However, the CV curves of the VN/C (I) showed much bigger curve areas compared to those of VN/C (II), indicating a higher capacitance. As shown in Fig. 4b, the GCD curves of the samples were measured at a current density of 0.5 A g^−1^. VN/C (I) showed a linear and slightly asymmetric triangle shape resembling the characteristics of a normal double-layer capacitor and indicating satisfactory electrochemical reversibility. The discharging time required for the VN/C (I) sample was longer than that of VN/C (II), indicating the better capacitance of VN/C (I). Moreover, from the relevant calculations, the mass specific capacitances of VN/C (I) and VN/C (II) were 392.0 and 245.1 F g^−1^, respectively, at a current density of 0.5 A g^−1^. EIS tests were also performed over a frequency range of 0.01 Hz to 100.0 kHz (Fig. 4c). The impedance curves contained one semicircle at a high frequency and a linear feature at low frequency. In addition, the internal resistance of the VN/C (I) (0.55 Ω) electrodes, acquired from the intercept of the plots on the real axis, was much smaller than that of VN/C (II) (0.64 Ω). This indicated good infiltration of the electrolyte caused by the introduction of oxygen-containing functional groups. Because of the weakened conductivity, the diameters of the semicircles of the VN/C (I) samples were larger than that of VN/C (II). Moreover, the conductivity was measured using a 4-point probe resistivity measurement system (RTS-9), and values of 8.3 and 9.7 S cm^−1^ for VN/C (I) and VN/C (II), respectively, were obtained. These results agreed well with the smaller charge transfer resistance of VN/C (II) indicated by the EIS analysis. In addition, the plots of the VN/C (I) Warburg angle were higher than those of the VN/C (II), indicating that the abundant pore structure was beneficial for the diffusion of electrolyte ions and resulted in a small diffusion impedance. Figure 4d shows the specific capacitances of the samples at different current densities. When the current density increased from 0.5 to 30 A g^−1^, the capacitance retention values for VN/C (II) and VN/C (I) were 46.1 and 50.5%, respectively. Thus, in terms of comprehensive capability, the VN/C (II) electrode was shown to be more suitable for use in supercapacitors.

**Fig. 5:**
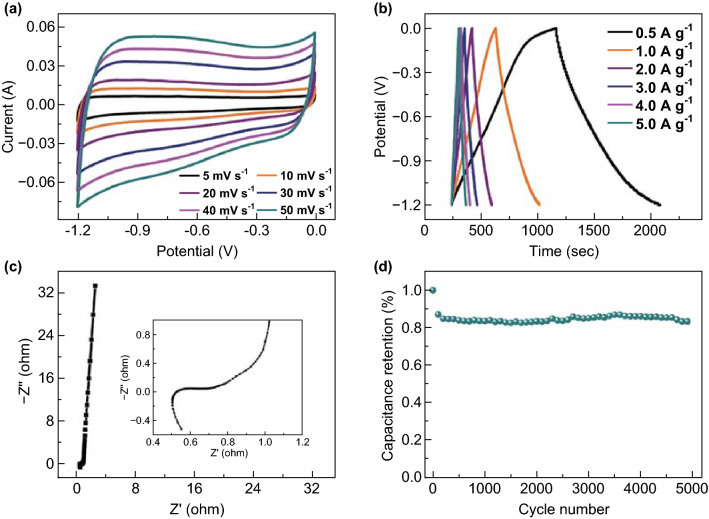
**a** CV, **b** GCD, **c** EIS curves, and **d** cycling performance at 2.0 A g^−1^ of the VN/C (I)

The electrochemical behavior of VN/C (I) at various current densities was also investigated. As shown in Fig. 5a, all CV loops were nearly quasi-rectangular in shape and almost no deformation was observed at high scan rates, indicating a small internal resistance. The low internal resistance was likely due to good wettability of the VN/C (I) electrode with electrolyte and its hierarchical porous structure, which was important for electron transport. The GCD curves (Fig. 5b) obtained at various current densities from 0.5 to 5 A g^−1^ exhibited a nearly linear and typical triangular symmetrical trend, demonstrating good electrode-reaction reversibility of the VN/C (I). The calculated specific capacitances were 392, 322, 295, 280, 276, 267 F g^−1^ at different current densities of 0.5, 1, 2, 3, 4, 5 A g^−1^, respectively. In addition, an excellent cycling stability of 83.5% was obtained at a current density of 2 A g^−1^ after 5000 cycles (Fig. 5d). Figure S6 shows the low- and high-resolution TEM images of VN/C (I) after 5000 cycles, which maintained their original morphology, indicating high stability. The TEM images show numerous small VN quantum dots homogeneously embedded in the porous carbon substrate. This explicitly indicates that the carbon matrix can prevent VN aggregation and simultaneously act as an active material for charge storage during the charging/discharging process. In conclusion, all the electrochemical results show convincingly that the prepared VN/C (I) is a promising electrode material.

**Fig. 6:**
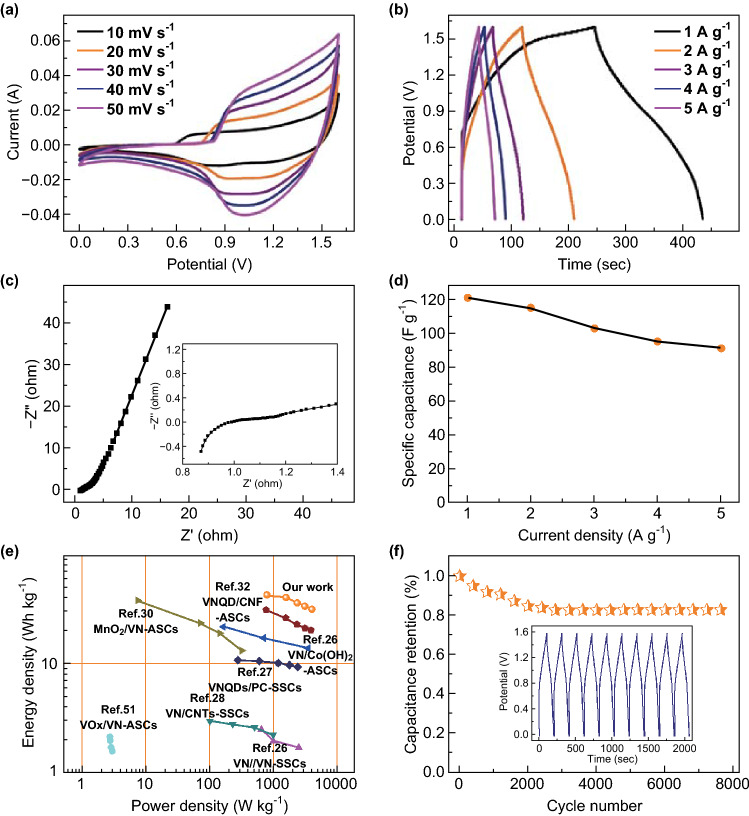
The electrochemical performance of the prepared hybrid device in 6.0 M KOH: **a** CV, **b** GCD, **c** EIS curves, and **d** specific capacitances of the device at different current densities. **e** Ragone plots compared with previously reported VN/C-based supercapacitors. **f** Cycling performance of VN/C (I) at 1.0 A g^−1^

To accurately assess the performance of the developed material for practical application, an asymmetric supercapacitor featuring a two-electrode system was assembled using VN/C (I) in 6 M KOH as the negative electrode and Ni(OH)_2_ as the positive electrode. Figure 6a shows the CV curves of the hybrid device over the voltage range of 0–1.6 V at various scanning rates between 10 and 50 mV s^−1^. In addition, a couple of wide oxidation reduction peaks at 1.0 V were observed, which were likely caused by the pseudocapacitive reactions related to the positive Ni(OH)_2_ and negative VN/C (I) electrodes. Figure 6b shows the linear potential–time relationship of the GCD curves of the hybrid device at different current densities from 1 to 5 A g^−1^ at working potential window of 1.6 V. The specific capacitance measured at the current density of 1 A g^−1^ was calculated to be 122 F g^−1^, and the retained capacitance was 91 F g^−1^ when the current density increased to 5 A g^−1^. As shown in Fig. 6c, the EIS of the hybrid device was tested in from 0.01 Hz to 100 kHz at room temperature. Notably, a small intercept at the real axis at approximately 0.87 Ω was observed, indicating a lower intrinsic resistance of the supercapacitors (SCs). Figure 6d shows a high rate performance where approximately 74.6% of specific capacitance was retained as the current density was raised from 1 to 5 A g^−1^. Moreover, the Ragone plots, as shown in Fig. 6e, showed the relationship between energy and power densities. The Ni(OH)_2_//VN/C (I) device showed an excellent energy density of 43 Wh kg^−1^ and high-power density of 800 W kg^−1^. From the comparison of power and energy densities in Fig. 6e and more detailed information in Table S3, the performance of the SCs was superior to related materials in the recently published papers. The cyclic stability of the SCs was determined by repetitive operation of the galvanostatic charging/discharging process (Fig. 6f). The Ni(OH)_2_//VN/C (I)-based SCs demonstrated excellent life cycle stability with 82.9% initial capacitance retention after 8000 cycles at a current density of 1.0 A g^−1^. Overall, the application potential of the prepared hybrid device was demonstrated by thorough characterization of all relevant electrochemical characteristics.

## Conclusion

In conclusion, the VN/C (I) design with interpenetrating carbon/VN networks, oxygen group-containing surfaces, and hierarchical porous structure was successfully fabricated for use as a supercapacitor electrode material. The advanced structure endowed VN/C (I) with a high specific surface area of approximately 523.5 m^2^ g^−1^ and excellent electrochemical behavior, including low resistance, good cyclic stability, and high specific capacitance. VN/C (I) presented a specific capacitance of 392.0 F g^−1^ at a current density of 0.5 A g^−1^ in 6.0 M KOH and a good rate capability with capacitance retention of 50.5% at 30 A g^−1^. Notably, the asymmetric device fabricated with Ni(OH)_2_//VN/C (I) exhibited a high energy density of 43.0 Wh kg^−1^ at a power density of 800 W kg^−1^, which only dropped to 32.3 Wh kg^−1^ at an increased power density of 4000 W kg^−1^. Moreover, excellent cycling stability (82.9%) was obtained at a current density of 1 A g^−1^ after 8000 cycles. This simple and novel strategy can be expanded to the synthesis of other hierarchical porous composite materials combining carbon-based and transition-metal oxide (nitride or sulfide) materials for numerous application in sensors, catalysts, gas separators, and other electrodes in hybrid supercapacitors.

## Electronic supplementary material

Below is the link to the electronic supplementary material.

Supplementary material 1 (PDF 874 kb)
