# Supplementary material for: A Novel Hierarchical Porous 3D Structured Vanadium Nitride/Carbon Membranes for High-performance Supercapacitor Negative Electrodes
Source: Nanomicro Lett. 2018 Jul 13;10(4):63. doi: 10.1007/s40820-018-0217-1 (PMC6199110; doi:10.1007/s40820-018-0217-1)
Supplement: Supplementary file 1 — Supplementary material 1 (PDF 874 kb) [file 40820_2018_217_MOESM1_ESM.pdf]

Supporting Information for

A Novel Hierarchical Porous 3 D Structure Vanadium Nitride/Carbon Membrane for high performance Supercapacitor Negative Electrodes

Yage Wu[1], Yunlong Yang[1], Xiaoning Zhao[1], Yongtao Tan[1], Ying Liu[1], Zhen Wang[1],

Fen Ran[1, 2,]

[1]State Key Laboratory of Advanced Processing and Recycling of Non-ferrous Metals, Lanzhou University of Technology, Lanzhou 730050, People's Republic of China

2School of Material Science and Engineering, Lanzhou University of Technology, Lanzhou 730050, Gansu, People's Republic of China

*Corresponding author. E-mail: ranfen@163.com; or ranfen@lut.cn (Fen Ran)

Supplementary Figures and Tables

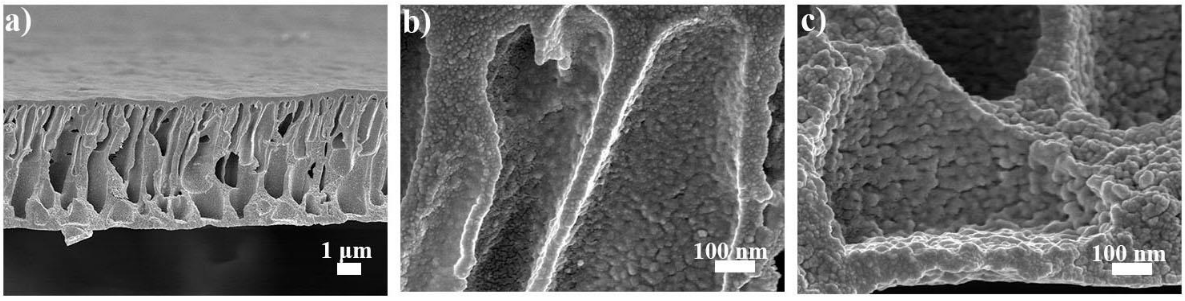

Fig. S1 SEM images: **a-c** the cross-section views of VN/C (II)

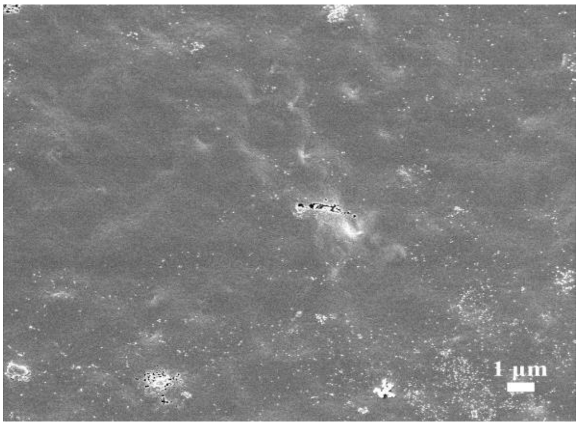

Fig. S2 The surface SEM view of VN/C (II)

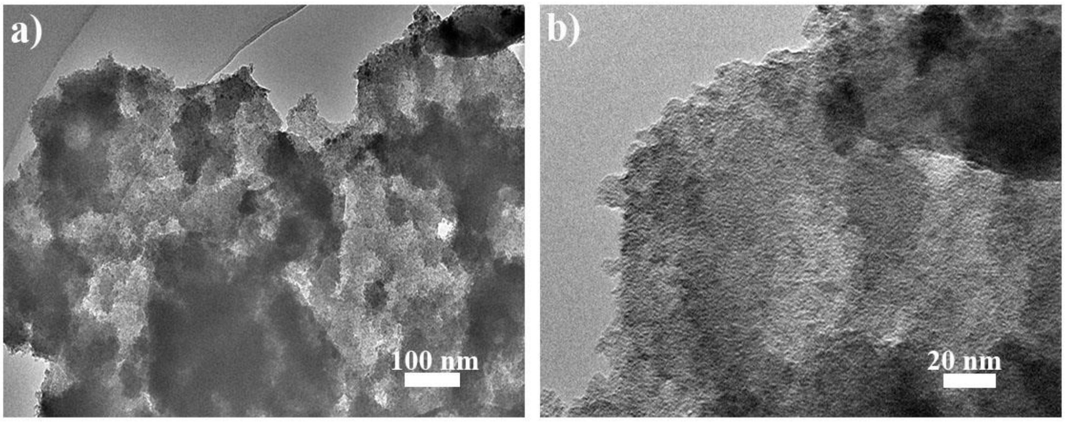

Fig. S3 The TEM images in different magnification multiples of VN/C (II)

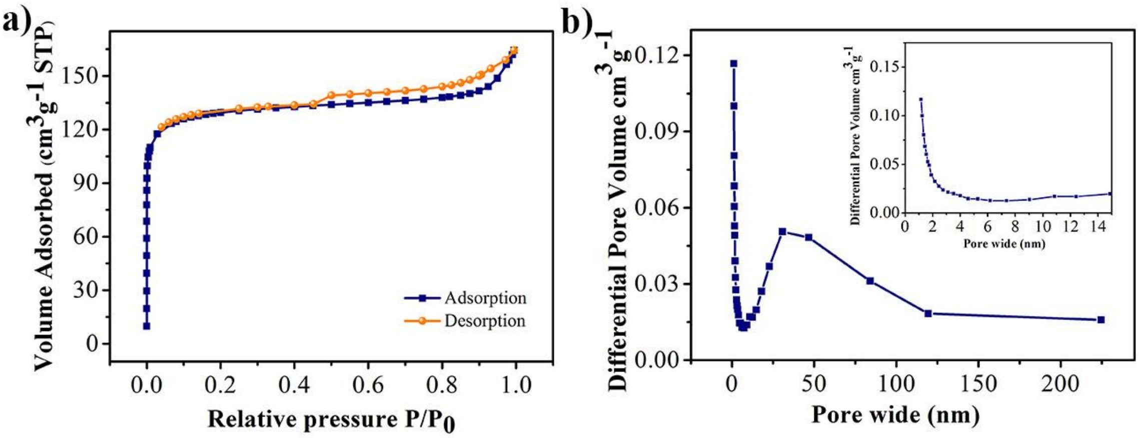

Fig. S4 a N_2 adsorption-desorption isotherms, and **b** pore size distribution of VN/C (II)

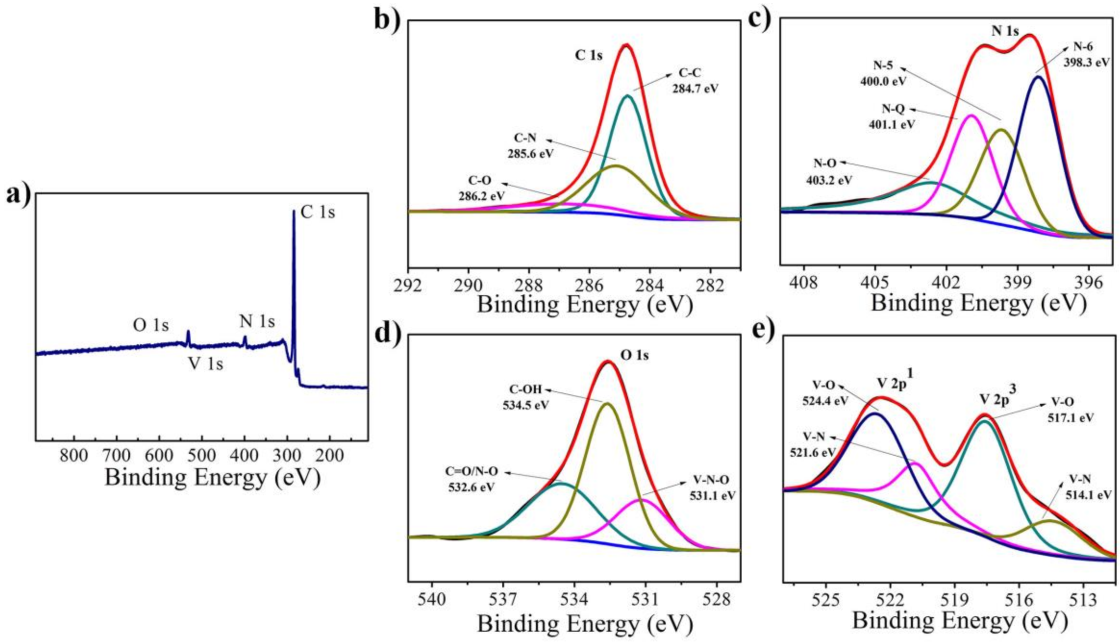

Fig. S5 X-ray photoelectron spectra of VN/C (II)

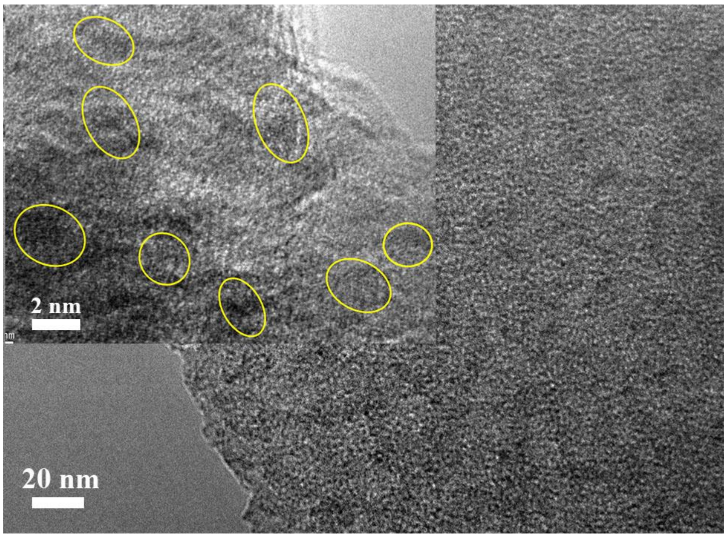

Fig. S6 TEM images of VN/C (I) after cycling

Table S1 BET surface areas and pore volumes of the samples

Samples	BET Surface Area (m^2 g^{-1})	t-Plot Micropore Area (m^2 g^{-1})	Total Pore Volume (cm^3 g^{-1})	Micropore Volume (cm^3 g^{-1})	Micropore Area Ratio
VN/C (II)	504.2	485.9	0.25	0.19	76.0 %
VN/C (I)	523.5	501.8	0.24	0.10	83.5 %

Table S2 Elemental analysis and XPS surface characterization of the VN/C (I) and VN/C (II)

**Sample**	**C at%**	**N at%**	**O at%**	**V at%**	**C-O at%**	**N-O at%**	**C-OH at%**
VN/C (I)	88.0	3.6	6.1	2.3	20.0	20.9	29.7
VN/C (II)	87.8	4.2	5.3	2.7	17.1	12.3	16.9

Table S3 The electrochemical performance of various VN-based electrodes reported in literature

Negative materials	Supercapacitors (SCs)	Electrolyte Solution	Specific capacitance of SCs	E_{max} (Wh kg^{-1})	P_{max} (W kg^{-1})	Voltage Window	References (year)
VN	VN//VN Symmetric SCs	aqueous 2 M KOH	17.5 F g^{-1} at 1 A g^{-1}	2.7	4800	0~1 V	**[1]** (2014)
VNQDs/PC	VNQDs/PC//VNQDs/PC Symmetric SCs	aqueous 6 M KOH	53.75 F g^{-1} at 0.5 A g^{-1}	10.75	3000	0~1.2 V	**[2]** (2016)
VN/CNTs	VN/CNTs//VN/CNTs Symmetric SCs	aqueous 6 M KOH	—	4	10000	0~1 V	**[3]** （2011）
AC	AC//V_2O_5·$0.6H_2O$ Asymmetric SCs	aqueous 0.5 M K_2SO_4	64.4 F g^{-1} at current rate of 2C	29.0	2000	0~1.8 V	**[4]** （2009）
VN-MWCNT	VN-MWCNT//MnO_2-MWCNT Asymmetric SCs	aqueous 0.5 M Na_2SO_4	86 F g^{-1} at 0.25 mA cm^{-2}	38.7	316.2	0~1.8 V	**[5]** （2014）
PCNS@VNNP	PCNS@VNNP//NiO Asymmetric SCs	aqueous 2 M KOH	75 F g^{-1} at 1 A g^{-1}	16	800	0~1.6 V	**[6]** （2016）
VNQD/CNF	VNQD/CNF//$Ni(OH)_2$ Asymmetric SCs	aqueous 6 M KOH	93.5 F g^{-1} at 1 A g^{-1}	31.2	3875	0~1.55 V	**[7]** （2017）
**VN/C (I)**	**VN/C-M // $Ni(OH)_2$ Asymmetric SCs**	**aqueous 6 M KOH**	**122 F g^{-1} at 1 A g^{-1}**	**43.0**	**4000**	**0~1.6 V**	**our work**

References

[1] R. Wang, X. Yan, J. Lang, A hybrid supercapacitor based on flower-like $Co(OH)_2$ and urchin-like VN electrode materials. J. Mater. Chem. A **2**, 12724-12732 (2014). https://doi.org/10.1039/C4TA01296H

[2] Y. Yang, L. Zhao, K. Shen, Y. Liu, X. Zhao, J. Wu, F. Ran, Ultra-small vanadium nitride quantum dots embedded in porous carbon as high performance electrode materials for

capacitive energy storage. J. Power Sources **333**, 61-71 (2016). https://doi.org/10.1016/j.jpowsour.2016.09.151

[3] C. Ghimbeu, E. Raymundo-Pi-ero, P. Fioux, Vanadium nitride/carbon nanotube nanocomposites as electrodes for supercapacitors. J. Mater. Chem. **21**, 13268-13275 (2011). https://doi.org/10.1039/c1jm11014d

[4] Q. Qu, Y. Shi, L. Li, $V_2O_5 \cdot 0.6\ H_2O$ nanoribbons as cathode material for asymmetric supercapacitor in K_2SO_4 solution. Electrochem. Commun. **11**, 1325-1328 (2009). https://doi.org/10.1016/j.elecom.2009.05.003

[5] Y. Su, I. Zhitomirsky, Hybrid MnO_2/carbon nanotube-VN/carbon nanotube supercapacitors. J. Power Sources **267**, 235-242 (2014). https://doi.org/10.1016/j.jpowsour.2014.05.091

[6] Y. Liu, L. Liu, L. Kong, L. Kang, F. Ran, Supercapacitor electrode based on nano-vanadium nitride incorporated on porous carbon nanospheres derived from ionic amphiphilic block copolymers & vanadium-contained ion assembly systems. Electrochim. Acta **211**, 469-477 (2016). https://doi.org/10.1016/j.electacta.2016.06.058

[7] Y. Wu, F. Ran, Vanadium nitride quantum dot/nitrogen-doped microporous carbon nanofibers electrode for high-performance supercapacitors. J. Power Sources **344**, 1-10. (2017). https://doi.org/10.1016/j.jpowsour.2017.01.095
